# Mental illness in metropolitan, urban and rural Georgia populations

**DOI:** 10.1186/1471-2458-13-414

**Published:** 2013-04-30

**Authors:** William C Reeves, Jin-Mann S Lin, Urs M Nater

**Affiliations:** 1 Public Health Surveillance and Informatics Program Office, Mail Stop E-33, Centers for Disease Control and Prevention, 1600 Clifton Road NE, Atlanta, GA 30333, USA; 2 Chronic Viral Diseases Branch, Division of High-Consequence Pathogens and Pathology, Mail Stop A-30, Centers for Disease Control and Prevention, 1600 Clifton Road NE, Atlanta, GA 30333, USA; 3 Department of Psychology, University of Marburg, Marburg, Germany

## Abstract

**Background:**

Mental illness represents an important public health problem. Local-level data concerning mental illness in different populations (e.g., socio-demographics and residence – metropolitan/urban/rural) provides the evidence-base for public health authorities to plan, implement and evaluate control programs. This paper describes prevalence and covariates of psychiatric conditions in Georgia populations in three defined geographic areas.

**Methods:**

Data came from the Georgia population-based random-digit-dialing study investigating unwellness and chronic fatigue syndrome (CFS) in Georgia populations of three defined geographic areas (metropolitan, urban, and rural). Respondents were screened for symptoms of fatigue, sleep, cognition, and pain at household screening interviews, and a randomly selected sample completed detailed individual phone interviews. Based on the detailed phone interviews, we conducted one-day clinical evaluations of 292 detailed interview participants classified as unwell with a *probable CFS (i.e. CFS-like;* a *functional somatic syndrome),* 268 classified as *other unwell, and* 223 *well* (matched to *CFS-like*). Clinical evaluation included psychiatric classification by means of the Structured Clinical Interview for DSM (SCID). To derive prevalence estimates we used sample weighting to account for the complexity of the multistage sampling design. We used 2- and 3-way table analyses to examine socio-demographic and urbanicity specific associations and multiple logistic regression to calculate adjusted odds ratios.

**Results:**

Anxiety and mood disorders were the most common psychiatric conditions. Nineteen percent of participants suffered a current anxiety disorder, 18% a mood disorder and 10% had two or more conditions. There was a significant linear trend in occurrence of anxiety or mood disorders from *well* to *CFS-like*. The most common anxiety disorders were post-traumatic stress disorder (PTSD) (6.6%) and generalized anxiety disorder (GAD) (5.8%). Logistic regression showed that lower education and female sex contributed significantly to risk for both PTSD and GAD. In addition, rural/urban residence and Hispanic ethnicity were associated with PTSD. We defined moderate to severe depression as Major Depressive Disorder or a Zung score >60 and logistic regression found lower education to be significantly associated but sex, age and urbanicity were not.

**Conclusions:**

Overall occurrence of anxiety and mood disorders in Georgia mirrored national findings. However, PTSD and GAD occurred at twice the published national rates (3.6 and 2.7%, respectively). State and local prevalence and associations with education, sex and urbanicity comprise important considerations for developing control programs. The increased prevalence of anxiety and mood disorders in people with a functional somatic syndrome (or *CFS-like* illness) is important for primary care providers, who should consider additional psychiatric screening or referral of individuals presenting with somatoform symptoms.

## Background

Mental illness is characterized by sustained abnormal alterations in thinking, mood or behavior associated with distress and impaired functioning [1]. Mental illness represents an important public health problem. According to the World Health Organization, mental illnesses account for more collective disability burden in developed countries than any other group of illnesses, including cancer and heart disease [2]. Published studies have reported that about one quarter of adults in the United States have a mental illness, and that about 46% will develop one during their lifetime [3,4]. Mental illness is estimated to cost the United States at least $300 billion annually, including disability benefits of approximately $24 billion [5], health care expenditures of $100 billion [6] and lost earnings and wages of $193 billion [7].

Disability and the burden imposed by mental illness also reflect associations with comorbid conditions. For example, depression is significantly associated with cardiovascular disease, diabetes, obesity, asthma, epilepsy, and cancer and with health risk behaviors such as alcohol abuse [8-11]. People with mental illness also have lower rates of access to and utilization of health care, poor chronic disease treatment compliance and a higher risk of adverse outcomes [12-15]. Finally, those with mental illness use tobacco more than the general population [16], and their rates for both intentional (e.g., homicide, suicide) and unintentional (e.g., motor vehicle) injuries are two to six times higher than in the general population [17,18].

Various treatment and management interventions can reduce morbidity associated with specific mental illnesses and these interventions can be used by public health mental illness control programs. Public health control strategies for mental illness aim to reduce its prevalence, improve the clinical course, and reduce attendant impairment. Allocation of state and local resources for these programs must consider metropolitan, urban and rural population’s differences in burden of illness. In addition, risk of various mental illnesses varies between women and men, by age and by socio-economic status. Providing mental illness services requires outreach, clinical training and staffing appropriate to each illness and setting. Finally, some categories of mental illness disproportionately affect disadvantaged groups (e.g., racial ethnic minorities, low education, below poverty level) and this must be considered as part of resource allocation. Public health officials need accurate population specific information concerning the occurrence of mental illness in order to design control strategies, implement them and evaluate their effectiveness.

Obtaining this information requires population surveys to estimate the prevalence of specific mental illnesses and associated risk factors. Mood and anxiety disorders frequently present with unexplained somatic symptoms [19]. Such functional somatic syndromes commonly include complaints of fatigue, cognitive dysfunction, sleep disturbances and diffuse bodily pain [20-22]. One cost effective strategy for surveying mental illness uses an initial screening to identify those likely to have the illness followed by clinical evaluation of those who screen positive. This study compares detection of DSM-IV Axis I disorders [23] by means of the SCID in people screened as having a functional somatic syndrome and randomly selected people who did not meet screening criteria.

## Methods

### This study aimed to investigate the prevalence and correlates of major psychiatric disorders in metropolitan, urban, and rural Georgia populations

#### Data source

Data came from a study of unwellness and chronic fatigue syndrome (CFS) conducted between September 2004 and July 2005 in metropolitan, urban, and rural populations of Georgia [24]. The source study was approved by the Institutional Review Board of the Centers for Disease Control and Prevention (CDC) and affiliated institutions. All subjects gave written informed consent for participating in the study.

The source study included residents of three Georgia populations: 1) metropolitan Atlanta (Fulton and DeKalb Counties); 2) urban (Macon, Bibb County and the city of Warner Robins in adjacent Houston County); and 3) rural (10 counties surrounding Bibb – Houston (excluding Warner Robins), Baldwin, Bleckley, Crawford, Jones, Macon, Monroe, Peach, Twiggs, and Wilkinson). The study was carried out in three stages: 1) household screening telephone interview; 2) individual detailed telephone interview; and 3) clinical evaluation.

#### Household screening telephone interview

Sampling methodology and subjects have been described in detail [24]. In brief, a screening interview, with an adult household informant, (response rate 79%) elicited demographic and health status of each household resident aged 18 to 59 and identified them as *unwell* or *well*. We defined *unwell* as the informant noting current fatigue, cognitive impairment, unrefreshing sleep, or musculoskeletal pain in a household resident during the last month. We defined household residents as *well* if the informant had not noticed any of these symptoms during the last month. A total of 12,089 adults were selected to participate in a detailed telephone interview: 3,851 residents identified as *unwell with fatigue,* 5,122 *unwell but not fatigued*, and 3,116 *well*.

#### Individual detailed telephone interview

We conducted detailed computer-assisted telephone interviews with 5,630 household residents aged 18 to 59 identified by the informant as *well* or *unwell*. Seven people were later confirmed as age-ineligible for the detailed telephone interview. The detailed interview covered socio-demographic characteristics, current symptoms and their duration, and diagnosis of medical and psychiatric conditions considered exclusionary for clinic participation. Exclusionary conditions would confound associations of interest to the source study (of chronic fatigue syndrome) or could pose a risk or inconvenience for clinic participation. One thousand six hundred and nine (28.6%) reported at least one such condition. Medical exclusions were the most common; morbid obesity (Body Mass Index > =40 kg/m^2^; 6%), sleep apnea (5%), or recent pregnancy (5%). One hundred sixty-five (2.9%) reported a psychiatric diagnosis; these included 72 (1.3%) reporting a diagnosis of bipolar disorder, 49 (0.9%) substance or alcohol abuse, 30 (0.5%) schizophrenia, seven (0.1%) anorexia or bulimia, and seven (0.1%) other [25].

Based on the detailed interview data, we classified respondents who reported severe fatigue of at least six months duration and was accompanied by problems sleeping, cognitive difficulties and diffuse pain as *CFS-like*, which for the present analysis we considered a *functional somatic syndrome (*with high likelihood of a DSM-IV axis-I condition) [19,20,26]. We classified those who reported unwellness ≥ 6 months that did not meet *functional somatic syndrome* criteria as *other unwell.* We classified remaining respondents as *well*.

#### Clinical evaluation

We invited all 469 individuals with a *functional somatic syndrome* (i.e., *CFS-like*)(and no exclusion) to participate in a one-day clinical evaluation, and 292 (62%) completed. We invited 505 randomly selected *other unwell* people to participate, and 268 (53%) completed. Finally, 223 randomly selected *well* individuals, frequency matched to the *functional somatic syndrome* participants by sex, race, age (+/− 3 years), and residential area, completed the one-day clinical evaluation.

During clinical evaluations a licensed nurse practitioner or physician assistant reviewed current medications, past medical history, and completed a review of systems. Participants then underwent a complete standardized physical examination by a physician and provided blood and urine specimens for routine clinical laboratory testing [24]. Licensed and specifically trained psychiatric interviewers administered the research version of the Structured Clinical Interview for DSM-IV (SCID) [27]. Psychologists on the CDC research team performed quality control checks of SCID interview results and monitored interviewers’ technique on a regular basis. Raw SCID data was recorded as: 1 = Absent; 2 = Subthreshold and 3 = Threshold or Present. We identified the occurrence of #3 (*Threshold or Present*) for both current episode (past month) and lifetime occurrence. Clinic participants also completed the Self-Rating Depression Scale (SDS) [28], a 20-item questionnaire that quantifies the severity of current depression. We did not assess somatoform disorders identified by the SCID in this study because our objective was to use the telephone functional somatic syndrome screen to detect specific Axis-I disorders.

Analyses considered the following DSM-IV axis I diagnostic classes [23]: 1) mood disorders, which included major depressive disorder, dysthymia, depression not otherwise specified (NOS) and mood disorder due to a general medical condition (GMC); 2) anxiety disorders, comprised of panic disorder, generalized anxiety disorder(GAD), agoraphobia, specific phobia, social phobia, post-traumatic stress disorder (PTSD), obsessive-compulsive disorder (OCD), anxiety disorder due to general medical condition (GMC), substance-induced anxiety disorder , and anxiety NOS; 3) substance use disorders; and 4) adjustment disorder. Because not all people with severe depression meet DSM-IV criteria for major depressive disorder, we defined an additional category current moderate to severe depression as individuals diagnosed as major depressive disorder with the SCID or having a Zung Self Rating Depression score > 60 [28].

#### Sampling weights

Prevalence estimates and statistical analyses used weighted data. The purpose of sampling weights is to account for the complexity of the multistage sampling design, non-response, and post-stratification. We generated two main sampling weights: one for the individuals who complete detailed interviews (termed interview sampling weights) and the other for the subset who complete clinical evaluations (termed clinical sampling weights). The details about these two sampling weights have been described elsewhere [24]. Sampling weights are assigned to each person based on the number of people they represent within the 2000 U.S. Census non-institutionalized civilian population. Sampling weights incorporated a further adjustment for clinical evaluation nonresponse and reflected the probability of selecting chronically unwell people for clinical-evaluation. Because “well” subjects were selected for clinical evaluation based on matching to those with a functional somatic syndrome, we estimated their sampling rates based on the response-rate adjusted sampling rates for the underlying sample of completed telephone interviews. Briefly, we estimated pseudo-weights, by three geographic strata, for the matched sub-sample based on observed demographic characteristics (age, sex, race (Black, White/Other) and the known sampling weights for similar individuals in the CFS-like sample. We weighted the 3 sampling strata in proportion to their size.

#### Statistical analyses

This analysis only concerned the clinical sample of 765 subjects who had complete information from SCID interviews. We used the survey procedures of SAS Version 9.2 (SAS Institute Inc, Cary, NC) for all analyses to account for the complex sampling design (sampling weights) to calculate weighted estimates. We report weighted percentages of psychiatric disorder occurrence and their standard errors. We used 2- and 3-way table analyses to examine their association with geographic strata and other socio-demographic strata. We used logistic regression to estimate adjusted odds ratios (AORs) for categories of psychiatric disorders, controlling for socio-demographics. Significance level was 0.05 for two-sided tests.

## Results

### Clinic participant characteristics

Table 1 summarizes characteristics of all 782 clinic participants by unweighted frequency and weighted percentage.

**Table 1:** Clinic Participant Characteristics (n = 782)

	**n**^**a**^	**Weighted %**
**Urbanicity**		
Metropolitan	132	76.2
Urban	267	11.1
Rural	383	12.8
**Sex**		
Female	597	58.1
Male	185	42.0
**Race**		
White	550	51.6
Black	197	35.0
All Other	35	13.5
**Ethnicity**		
Hispanic	20	2.3
Non-Hispanic	762	97.7
**Age**		
18-29	94	28.2
30-39	146	18.0
40-49	283	27.1
50-59	259	26.8
**Education**		
< High School	205	14.3
High School +/−College	318	31.8
> College	257	53.9
**Income**		
< $20,000	176	24.8
$20,000-$40,000	127	22.7
>$41,000	450	52.6
**Poverty**		
<$20,000	176	24.8
>$20,000	577	75.2

^a^ Not all 782 answered all questions concerning education, income or poverty status and 17 did not complete the SCID.

### Overall occurrence of psychiatric conditions

#### Specific psychiatric conditions

Seven hundred sixty-five clinic participants had complete SCID data and we identified at least one current DSM-IV psychiatric disorder in 42% of them (Table 2). Anxiety and mood disorders represented the most commonly identified current psychiatric illnesses and occurred at similar rates (19.4 and 18.5 percent, respectively). The most common anxiety disorders were post-traumatic stress disorder (PTSD) (6.6%) and generalized anxiety disorders (GAD) (5.8%) followed by a specific phobia (4%). Just over 1% had social phobia and obsessive compulsive disorder. Panic or agoraphobia and other anxiety disorders were rare. As expected, depression NOS and major depressive disorder dominated the mood disorders. More (45.8%) participants had suffered a lifetime mood disorder than a lifetime anxiety disorder (31.7%).

**Table 2:** Weighted prevalence and 95% confidence intervals (CI) of current and lifetime DSM-IV psychiatric conditions in selected Georgia counties

**Current DSM-IV Conditions**					**Lifetime DSM-IV Conditions**				
	**n**	**Weighted percent**	**Lower CI**	**Upper CI**		**n**	**Weighted percent**	**Lower CI**	**Upper CI**
**Anxiety Disorders**	287	19.4	12.7	26.2	**Anxiety Disorders**	374	31.7	19.8	43.5
Anxiety NOS	77	7.1	3.2	11.0	Anxiety NOS	86	14.5	2.9	26.0
PTSD	115	6.6	3.8	9.3	PTSD	216	9.9	6.3	13.5
GAD	98	5.8	2.5	9.1					
Specific Phobia	67	4.0	1.8	6.2	Specific Phobia	90	6.6	2.9	10.3
Social Phobia	35	1.2	0.5	2.0	Social Phobia	51	2.5	1.0	4.0
OCD	28	1.2	0.1	2.3	OCD	41	2.3	0.8	3.8
Panic	26	1.0	0.3	1.8	Panic	66			
Agoraphobia	12	0.8	0.00	1.6	Agoraphobia	21	1.5	0.3	2.6
**Mood Disorders**	139	18.5	5.4	31.7	**Mood Disorders**	424	45.8	32.0	59.6
Depression NOS	22	10.0	0.0	23.6	Depression NOS	72	21.8	6.0	37.6
Major Depressive Disorder	101	7.3	3.2	11.5	Major Depressive Disorder	350	25.2	16.9	33.5
Dysthymia	16	1.3	0.01	2.6					
**Adjustment Disorders**	39	3.2	1.0	5.3					

#### Multiple conditions

Among clinic participants with an anxiety or a mood disorder, most (19.6%) had one psychiatric condition, 7.4% had two conditions, 2.0% three, 1.1% four, and we identified five or more conditions in 0.3%. Anxiety disorders were the most common; among participants with only one condition, as 27.4% had Anxiety NOS, 14.9% had PTSD, 14.3% major depressive disorder, 12.1% GAD and 11.4% Specific Phobia. PTSD and GAD were most common among those with two disorders (44.4% and 42.0%, respectively. This general order also occurred for those with three, four, or ≥ five conditions (Table 3).

**Table 3:** Occurrence of one, two, three, four and ≥ five DSM-IV conditions

**One DSM-IV Condition**	**Weighted %**	**Two DSM-IV Conditions**	**Weighted %**	**Three DSM-IV Conditions**	**Weighted %**	**Four DSM-IV Conditions**	**Weighted %**	**≥Four DSM-IV Conditions**	**Weighted %**
Anxiety NOS	27.4	PTSD	44.4	PTSD	62.5	MDD	66.7	PTSD	92.9
PTSD	14.9	GAD	42.0	GAD	59.2	Specific Phobia	61.9	Specific Phobia	92.3
MDD	14.3	MDD	38.3	MDD	53.1	PTSD	61.9	GAD	64.3
GAD	12.1	Specific Phobia	18.8	Social Phobia	22.9	Social Phobia	52.4	Hypochondriasis	64.3
Specific Phobia	11.4	Anxiety NOS	18.5	Specific Phobia	20.4	GAD	33.3	MDD	57.1
Depression NOS	5.8	Depression NOS	8.6	Anxiety NOS	18.4	Panic	28.6	Panic	50.0
Dysthymia	4.7	OCD	7.4	OCD	16.7	Anxiety NOS	28.6	Social Phobia	50.0
OCD	3.4	Social Phobia	6.2	Panic	14.3	OCD	25.0	OCD	21.4
Panic	1.7	Agoraphobia	4.9	Dysthymia	8.5	Hypochondriasis	19.1	Anxiety NOS	21.4
Hypochondriasis	1.7	Panic	3.7	Agoraphobia	8.2	Dysthymia	10.0	Dysthymia	14.3
Social Phobia	1.7	Mood – GMC	2.5	Depression NOS	6.1	Agoraphobia	9.5	Depression NOS	14.3
Mood – GMC	0.6	Dysthymia	1.3	Mood – GMC	6.1	Depression NOS	4.8	Agoraphobia	14.3
Agoraphobia	0.6	Mood–Substance	1.2	Hypochondriasis	4.1				
		Anxiety-GMC	1.2	Anxiety–Substance	2.0				
		Anxiety–Substance	1.2						

Weighted % – % of those with 1, 2, 3, etc. conditions that have condition in that row.

NOS – Not Otherwise Specified.

PTSD – Post-Traumatic Stress Disorder.

MDD – Major Depressive Disorder.

GAD – Generalized Anxiety Disorder.

OCD – Obsessive-Compulsive Disorder.

GMC – accompanying a general medical condition.

Substance – accompanying a substance abuse disorder.

The remainder of this paper concerns current anxiety and mood disorders identified during the clinic evaluation. Both current anxiety and current mood disorders were significantly more common among *functional somatic syndrome* participants and there was a significant linear trend in occurrence increasing from *Well* to *Unwell* to *functional somatic syndrome* (p < .001) (Table 4).

**Table 4:** Association of current anxiety and mood disorders by telephone interview classification as functional somatic syndrome, other unwell and well

**Current Condition**	**Sample Category for Clinic Participation**						
	***Somatic Syndrome***	**Wtd %**	***Other Unwell***	**Wtd %**	***Well***	**Wtd %**	**Waldχ2 Statistics**
Any of Anxiety Disorders							26.4
No	136	45.8%	170	82.0%	180	88.8%	
Yes	156	54.2%	98	18.0%	42	11.2%	
Any of Mood Disorders							24.5
No	195	61.9%	229	79.4%	213	95.9%	
Yes	97	38.1%	39	20.6%	9	4.1%	

χ2 test for Sample category and any current Affective disorder p <0.001.

χ2 test for Sample category and any current Mood disorder p <0.001.

### Anxiety and mood disorders

#### Prevalence

Overall, weighted prevalence of current anxiety disorders (19.4%) and current mood disorders (18.5%) were similar. Prevalence varied considerably by demographic features across the metropolitan, urban, and rural samples (Tables 5 and 6).

**Table 5:** Weighted prevalence and 95% confidence intervals (CI) of any current anxiety disorder by demographic features in the Metropolitan, Urban and Rural populations

	**Metro**		**Urban**		**Rural**	
	***Wtd %***	***95% CI***	***Wtd %***	***95% CI***	***Wtd %***	***95% CI***
**Overall**	9.9	4.3-15.51	28.7	19.2-38.1	26.9	19.8-34.0
**Sex**						
Female	19.8	10.4-29.1	42.6	31.4-53.8	43.4	33.7-53.1
Male	7.5	0-17.7	26.5	9.9-43.1	22.2	11.6-32.8
**Race**						
Other	6.4	0-17.7	34.0	0-78.2	45.9	11.9-79.9
Black	17.9	6.6-29.2	35.0	20.5-49.4	37.8	19.4-56.2
White	15.3	3.0-27.6	36.2	22.9-49.4	31.3	23.3-39.3
**Ethnicity**						
Hispanic	23.7	0-60.7	35.1	0-85.9	50.1	6.6-93.5
Non-Hispanic	14.5	6.8-22.2	35.7	25.8-45.5	33.0	25.4-40.5
**Age**						
18-29	5.4	0-12.0	40.3	16.4-64.1	43.8	21.4-66.2
30-39	10.9	0.8-20.9	39.9	22.1-57.8	49.6	31.8-67.4
40-49	23.4	3.6-43.3	25.8	14.6-36.9	20.4	11.8-29.1
50-59	19.7	3.0-36.3	39.0	16.2-61.8	29.3	17.6-41.0
**Education**						
< HS	38.6	2.6-74.7	37.8	21.3-54.3	35.3	20.3-50.3
HS-College	11.6	0.6-22.7	41.5	25.1-57.9	35.4	24.1-46.7
> College	12.3	3.7-20.9	24.5	11.2-37.7	25.8	15.1-36.5
**Income**						
< $20,000	19.4	1.3-37.6	38.5	21.5-55.5	28.0	12.4-43.6
$20-$40 K	8.7	0-24.4	55.9	35.8-76.0	49.2	27.2-71.2
>$41,000	14.3	4.7-23.8	26.1	12.6-39.6	29.0	20.4-37.6
**Poverty**						
>$20,000	12.5	4.3-20.8	35.0	23.4-46.7	33.4	24.8-42.0
<$20,000	19.4	1.3-37.6	38.5	21.5-55.5	28.0	12.4-43.6

**Table 6:** Weighted prevalence and 95% confidence intervals (CI) of any mood disorder by demographic features in the Metropolitan, Urban and Rural populations

	**Metro**		**Urban**		**Rural**	
	***Wtd %***	***95% CI***	***Wtd %***	***95% CI***	***Wtd %***	***95% CI***
**Overall**	20.6	3.6-37.7	14.3	8.6-19.7	9.5	5.5-13.6
**Sex**						
Female	13.6	5.7-21.6	18.3	10.5-26.0	13.3	6.7-19.8
Male	30.8	0-68.4	9.0	1.2-16.9	5.4	1.3-9.5
**Race**						
Other	63.6	14.9-100.0	0	0-0	7.8	0-16.9
Black	10.5	2.8-18.3	16.7	6.8-26.7	16.8	4.6-29.0
White	13.2	1.9-24.5	13.7	6.4-21.0	7.0	3.4-10.7
**Ethnicity**						
Hispanic	39.7	0-90.1	0.0	0-0	2.2	0-6.9
Non-Hispanic	20.2	2.74-37.6	14.7	8.8-20.5	9.6	5.5-13.7
**Age**						
18-29	32.4	0-77.9	13.5	0-27.4	13.4	0-28.1
30-39	12.6	0.01-25.2	17.7	4.7-30.7	17.1	5.5-28.7
40-49	23.6	3.5-43.7	14.4	5.4-23.3	4.0	1.5-6.6
50-59	9.0	0.6-17.5	11.6	1.6-21.6	7.7	2.2-13.3
**Education**						
< HS	19.7	0-49.8	25.8	11.7-39.9	11.6	3.9-19.3
HS-College	44.8	3.5-86.1	10.7	3.5-18.0	10.0	3.6-16.4
> College	10.3	2.6-17.9	8.8	0-18.1	5.7	0.1-11.3
**Income**						
< $20,000	13.7	0-29.2	27.9	12.1-43.8	7.7	2.2-13.3
$20-$40 K	54.9	11.7-98.2	18.2	4.5-32.1	16.9	4.2-29.5
>$41,000	9.1	2.4-15.9	6.7	1.5-11.8	7.5	2.1-12.9
**Poverty**						
<$20,000	13.7	0-29.2	27.9	12.1-43.8	7.7	2.2-13.3
>$20,000	23.6	1.1-46.1	10.1	4.5-15.7	9.6	4.6-14.6

#### Adjusted odds ratios

To adjust for the main effects of socio-demographics, we calculated adjusted odds ratios for any current anxiety disorder and for any current mood disorder (Additional file 1: Table S1). Anxiety disorders were significantly more common among urban residents. Mood disorders were significantly less common among residents of the sampled rural counties and significantly more common among those who had attended high school or college.

### Anxiety disorders

#### Prevalence of specific anxiety disorders

Anxiety disorders encompass a variety of conditions with different clinical manifestations and reflect different socio-demographic associations. PTSD (6.6%) and GAD (5.8%) were the most common anxiety disorders identified in the Georgia study, and their occurrence varied between metropolitan, urban, and rural populations according to socio-demographic characteristics (Additional file 1: Tables S2 and S3).

#### Adjusted odds ratios of PTSD and GAD

We included socio-demographics variables in multiple logistic regression models to calculate adjusted odds ratios for PTSD and for GAD. Both were significantly more common among women and those with less than a high school education. PTSD was also significantly more common among rural and urban residents and Hispanics (Additional file 1: Table S4).

### Moderate to severe depression

#### Prevalence of depression

Major depressive disorder and depression NOS comprised the most common mood disorders detected by the SCID. To better characterize the population of depressed people in Georgia, we explored characteristics of current moderate to severe depression, which affected 7.39% of the Georgia population. Additional file 1: Table S5 presents weighted prevalence of current moderate to severe depression by demographic features in the metropolitan, urban, and rural populations. Women living in the urban strata were significantly more likely than men to be identified with moderate to severe depression and less than high school education was significantly associated in the rural population.

#### Adjusted odds ratio of moderate to severe depression

As with anxiety disorders, we included socio-demographic variables in multiple logistic regression models to calculate adjusted odds ratios for moderate to severe depression (major depressive disorder identified by the SCID or a Zung Self-Rating Depression Scale score > 60). Those with less than a high school education were significantly more likely to suffer current moderate to severe depression, Additional file 1: Table S6.

## Discussion

Current anxiety (19.4%) and mood disorders (18.5%) were prevalent in residents of the selected Georgia counties. Overall, these findings agree with published U.S. national-level surveys. The 2001 to 2003 NCS-R, the most comprehensive national study, used the Composite International Diagnostic Interview (CIDI) [29-31] and reported 18.2% of U.S. adults had an anxiety disorder and 19.5% a mood disorder [32]. Occurrence of major depressive disorder in the selected Georgia counties was similar to NCS-R national estimates (7.3% and 6.7%, respectively) [32]. Most of those with a mental illness detected in our study (19%) suffered only one, 7.4% had two psychiatric conditions, 2% had three, and 1.4% four or more, which also mirrors NCS-R reported findings; 14.4% of people with a psychiatric disorder had only one, 5.9% had two, and 5.9% three or more [32]. However both order and magnitude of specific anxiety disorder prevalence in our Georgia population sample differed markedly from 2001 to 2003 NCS-R national estimates. In Georgia, PTSD (6.6%) and GAD (5.8%) were the most common anxiety disorders followed by specific phobia (4.0%) and social phobia (1.2%); while, specific phobia (8.7%), social phobia (6.8%), PTSD (3.6%) and GAD were the most common anxiety disorders reported from NCS-R (2.7%) [32]. This difference in our findings from Georgia and NCS-R highlights that national surveys may not reflect category-specific occurrence of mental illness in individual states. For example, a recent report indicates depression and psychological distress are more common in the southeastern states than the nation as a whole [33]. However, it is also possible that this variation in prevalence estimates may be due to methodological differences between our study and the NCS-R, such as using a state-specific sample rather than a national sample and the SCID rather than CIDI as a diagnostic tool.

As we hypothesized, both current anxiety and current mood disorders were significantly more common among people identified by telephone interview as unwell (*functional somatic syndrome* or *other unwell*). Fifty three percent of *functional somatic syndrome* participants had a current anxiety disorder and 32% a current mood disorder; among those classified as *well* only 18.9% had an anxiety disorder and 3.7% a mood disorder. The increased prevalence of anxiety and mood disorders in people with a functional somatic syndrome is important for primary care providers, who should consider additional psychiatric screening or referral of individuals presenting with somatoform symptoms. This association also has implications for psychiatric nosology and development of DSM-V [19,22]. As noted in a recent review by Lieb et al. [34], there is a remarkable lack of data evaluating occurrence of anxiety and mood disorders among people with a functional somatic syndrome*.* Research surveys for mental illness should consider screening for *functional somatic syndromes* to help resolve questions of nosology and surveillance systems intended for public health use should also measure *functional somatic syndromes* as part of screening for anxiety and mood disorders.

In our Georgia study sample, anxiety and mood disorders varied considerably according to socio-demographic factors among metropolitan, urban and rural samples. Our study found no significant difference among the three geographic areas in the prevalence of having any current mood disorder, whereas previous U.S. studies have reported a slight but significantly higher prevalence of depression in rural areas than metropolitan area [35,36]. Future work should investigate whether there may be unique aspects of mental health treatment or access to treatment in urban and rural Georgia that are associated with this non-significant effect. On the contrary, for current anxiety disorders, our study results showed that there was a significantly higher prevalence in urban and rural areas than that in the metropolitan area. Logistic regression modeling of PTSD and GAD (the most common anxiety disorders) found both significantly associated with female sex and education less than high school. PTSD was also significantly associated with rural and urban residence but residence and ethnicity were not associated with GAD. This finding might indicate more limited availability of and referral to treatment opportunities for those living in rural and urban locations who have encountered traumatic events. A recent study has shown that veterans with PTSD had significantly fewer treatment visits when living in rural areas than those living in bigger cities [37]. Another study observed that referrals after a natural disaster were markedly higher in large cities (Atlanta among them) compared to urban locations [38]. Logistic regression modeling of moderate to severe depression showed less than high school education as the most significant factor and no significant association with sex or urbanicity. These findings add to the evidence base that should be considered when designing mental illness control programs. For both anxiety and mood disorders, messaging and education must consider educational attainment. In Georgia, programs for PTSD and GAD should also consider outreach to gynecologists, who often serve as women’s primary care providers. Finally, in Georgia the association of urban and rural residence with PTSD should be considered when developing clinical services and messaging for these segments of the population.

To our knowledge, CDC’s Behavioral Risk Factor Surveillance System (BRFSS) comprises the only population survey of depression in Georgia against which to compare our findings. In 2006 and 2008 BRFSS conducted random digit dial telephone interviews with adults and estimated that 9.0% were depressed during the last 2 weeks, as reflected by a PHQ-8 score >10 [33], which is similar to our finding that 9.8% of the selected Georgia counties’ study population suffered moderate to severe depression during the last month. BRFSS has also estimated that 3.4% of Georgia adults had major depression, which is quite different from our estimated 7.3% prevalence of major depressive disorder in the selected Georgia counties. This most likely occurred because the PHQ-8 screens for depression in the last two weeks and does not identify DSM-IV major depressive disorder as stringently measured by means of the SCID (at least two weeks during the last month). BRFSS has not published results concerning factors associated with depression in Georgia against which to compare our present findings (e.g., associations with age, sex, race/ethnicity, metropolitan/urban/rural residence, education, or household income). Unfortunately, BRFSS does not measure other categories of mental illnesses, such as anxiety disorders, which our survey found to be as common and disabling as depression and which also reflected different associations with socio-demographic variables than did depression.

### Strengths and limitations

One of the strengths of this study is the use of a large sample identified from metropolitan, urban, and rural Georgia populations through phone interviews and clinical evaluations. In addition, this study used the SCID as a diagnostic tool for psychiatric disorders evaluated in the paper.

There are two major limitations of our study. Most importantly, we used Fulton and DeKalb counties to represent metropolitan Georgia, Macon and Warner Robins represented urban Georgia, and counties surrounding them represented rural Georgia. We chose this approach for logistical reasons. Fulton and DeKalb counties are the heart of Atlanta and meet US Department of Agriculture metropolitan population criteria [39]. However, 14 additional counties comprise the Atlanta metropolitan area and were not sampled. Macon (Bibb County) and Warner Robins are considered urban [39] but are not necessarily similar to other Georgia urban populations (e.g., Savannah, Athens and Rome). Due to cross-sectional data, we could not infer causal relationships between the psychiatric diagnoses and socio-demographic variables. The study also suffered from the non-coverage for institutionalized and non-English speaking populations. Finally, although our rural study counties fulfill USDA criteria for rural [39], their populations may differ from those of rural counties in the north Georgia mountains or southern coastal plain. Further analyses of the publically available BRFSS database may provide information concerning the effects of such possible differences on occurrence of depression.

Second, selection for clinical evaluation was not random, but was based on wellness status detected by the telephone interview. The source study was enriched in fatiguing illness and potentially identified high prevalence of undiagnosed psychiatric conditions. We attempted to adjust for clinic selection bias by weighting, which has attendant methodological limitations. Finally, reflecting sample size, some standard errors are large.

## Conclusions

This report was limited to describing occurrence of anxiety and mood disorders in different Georgia populations. Additional analyses will evaluate other factors known to be associated with these disorders and that the survey also measured (e.g., obesity, metabolic syndrome, diabetes, childhood abuse, access to and utilization of health care, and economic impact). We evaluated the 2004–2005 survey cohort again between 2007 and 2009; analyses will explore clinical course of these anxiety and mood disorders, their incidence, and their associations with socio-demographic variables and comorbid conditions. Effective public health initiatives to control mental illness could be better focused based on this knowledge. Research surveys such as ours are not optimal for routine public health surveillance. However, BRFSS exemplifies successful state/local surveillance that collects uniform, state-specific data concerning preventive health practices and risk behaviors associated with chronic disease, injuries and infectious diseases in the adult population. States use BRFSS data to identify emerging health problems, to establish and track health objectives, and to develop and evaluate public health policies and programs. For many states, BRFSS is the only source of timely, accurate state-based data on health-related behaviors. BRFSS surveillance data for Georgia and other states is publically available [40] and future analyses will explore similarities between our mental illness and associated risk factors identified in our research study and BRFSS data related to depression and psychological distress. Based on this, we will plan mental health specific surveillance appropriate for use by states.

Our findings may provide public health officials with accurate population specific information concerning the occurrence of mental illness. This will enable them to design control strategies, implement these strategies, and evaluate their effectiveness, especially in disproportionately affected disadvantaged groups (e.g., racial ethnic minorities, low education, below poverty level).

## Abbreviations

SCID: Structured Clinical Interview for DSM; PTSD: Post-Traumatic Stress Disorder; GAD: Generalized Anxiety Disorder; CDC: Centers for Disease Control and Prevention; GMC: General Medical Condition; OCD: Obsessive Compulsive Disorder; NOS: Not Otherwise Specified; AOR: Adjusted Odds Ratios; BRFSS: Behavioral Risk Factor Surveillance System

## Competing interests

The authors declare that they have no competing interests.

## Authors’ contributions

WCR, JML and UMN were involved in the conception, design and conduct of the study. WCR JML and UMN were involved in the analysis and interpretation of the data. WCR, JML and UMN wrote the manuscript. All authors have read and approved the final manuscript.

## Pre-publication history

The pre-publication history for this paper can be accessed here:

http://www.biomedcentral.com/1471-2458/13/414/prepub

## Supplementary Material

Additional file 1 The supplementary material includes the supplement Tables S1-S6 for the adjusted odds ratios for socio-demographic correlates of major psychiatric disorders in metropolitan, urban, and rural Georgia population. Click here for file
