# Supplementary material for: Mental illness in metropolitan, urban and rural Georgia populations
Source: BMC Public Health. 2013 Apr 30;13:414. doi: 10.1186/1471-2458-13-414 (PMC3654957; doi:10.1186/1471-2458-13-414)
Supplement: Additional file 1 — The supplementary material includes the supplement Tables S1-S6 for the adjusted odds ratios for socio-demographic correlates of major psychiatric disorders in metropolitan, urban, and rural Georgia population. [file 1471-2458-13-414-S1.docx]

**Table S1. Adjusted Odds Ratios for Current Anxiety and Current Mood Disorders**

|  | Current Anxiety Disorder | | Current Mood Disorder | |
| --- | --- | --- | --- | --- |
| **Urbanicity** | Adjusted OR | 95% CI | Adjusted OR ^b^ | 95% CI |
| Rural | 1.9 | 0.8-4.4 | **0.3** | **0.1-1.0** |
| Urban | **2.5** | **1.2-5.3** | 0.6 | 0.2-1.6 |
| Metro | Referent |  | Referent |  |
| **Age** |  |  |  |  |
| 18-29 | 0.6 | 0.2-1.7 | 1.3 | 0.2-10.2 |
| 30-39 | Referent |  | Referent |  |
| 40-49 | 1.2 | 0.5-3.1 | 3.1 | 0.5-19.4 |
| 50-49 | 1.6 | 0.7-3.7 | 0.9 | 0.2-4.9 |
| **Sex** |  |  |  |  |
| Female vs. Male | 2.5 | 0.9-7.2 | 0.7 | 0.2-2.1 |
| **Race/Ethnic** |  |  |  |  |
| Other | 0.4 | 0.1-2.3 | 5.5 | 0.9-34.6 |
| Black | 0.8 | 0.4-1.9 | 0.7 | 0.2-2.1 |
| White | Referent |  | Referent |  |
| **Hispanic vs. Non-Hispanic** | 3.6 | 0.5-24.9 | 1.3 | 0.1-14.7 |
| **Education** |  |  |  |  |
| < HS | 3.2 | 0.9-11.0 | 1.9 | 0.2-15.7 |
| HS-College | 1.8 | 0.3-3.8 | **4.2** | **1.4-12.8** |
| > College | Referent |  | Referent |  |
| **Poverty vs. Above Poverty** | 1.5 | 0.4-5.2 | 1.2 | 0.3-5.2 |

^a^ Adjusted odds ratios controlling for all other socio-demographic variables in the table.

^b^ Sample size of Hispanic group was small.

**Table S2. Weighted Prevalence and 95% Confidence Intervals (CI) of PTSD by Demographic Features in the Metropolitan, Urban and Rural Populations.**

|  |  | **Metro** |  | **Urban** |  | **Rural** |  |
| --- | --- | --- | --- | --- | --- | --- | --- |
|  |  | ***Wtd %*** | ***95% CI*** | ***Wtd %*** | ***95% CI*** | ***Wtd %*** | ***95% CI*** |
| **Overall** |  | 3.7 | 1.0-6.4 | **16.3** | **9.0-23.6** | **14.8** | **8.6-21.0** |
| **Sex** |  |  |  |  |  |  |  |
|  | Female | 6.2 | 1.9-10.4 | **23.2** | **12.4-34.0** | **18.8** | **10.4-27.2** |
|  | Male | 0 | 0-0 | 7.4 | 0.2-14.6 | 10.4 | 1.3-19.4 |
| **Race** |  |  |  |  |  |  |  |
|  | Other | 2.7 | 0-8.9 | 27.1 | 0-70.5 | 5.9 | 0-14.7 |
|  | Black | 6.9 | 0.5-13.3 | 13.4 | 4.8-22.0 | 15.6 | 1.2-20.0 |
|  | White | 1.7 | 0-3.8 | 17.4 | 6.9-27.9 | 14.8 | 7.8-21.7 |
| **Ethnicity** |  |  |  |  |  |  |  |
|  | Hispanic | 19.6 | 0-55.4 | 35.1 | 0-85.9 | 17.9 | 0-50.0 |
|  | Non-Hispanic | 3.3 | 0.8-5.9 | 15.8 | 8.5-23.1 | 14.7 | 8.4-21.0 |
| **Age** |  |  |  |  |  |  |  |
|  | 18-29 | 2.0 | 0-5.5 | 35.2 | 11.3-59.1 | 17.2 | 1.4-33.0 |
|  | 30-39 | 1.7 | 0-5.1 | 13.1 | 0.9-25.3 | 27.6 | 8.4-46.8 |
|  | 40-49 | 5.0 | 0-10.5 | 7.8 | 2.8-12.7 | 6.3 | 2.8-9.9 |
|  | 50-59 | 5.6 | 0-12.82 | 13.3 | 1.9-24.7 | 13.4 | 3.6-23.3 |
| **Education** |  |  |  |  |  |  |  |
|  | < HS | 16.1 | 0-36.3 | 24.6 | 10.3-38.9 | 18.7 | 5.5-31.9 |
|  | HS-College | 4.2 | 0-9.7 | 15.3 | 3.0-27.6 | 14.7 | 5.4-24.0 |
|  | > College | 1.6 | 0-3.2 | 10.0 | 0-20.5 | 9.9 | 2.3-17.6 |
| **Income** |  |  |  |  |  |  |  |
|  | < $20,000 | 9.4 | 0-19.3 | 18.6 | 5.7-31.6 | 15.6 | 1.3-20.0 |
|  | $20-$40K | 1.4 | 0-4.4 | 32.3 | 10.9-53.6 | 26.2 | 5.4-47.1 |
|  | >$41,000 | 2.2 | 0-4.9 | 8.3 | 1.7-14.9 | 11.8 | 5.3-18.3 |
| **Poverty** |  |  |  |  |  |  |  |
|  | >$20,000 | 1.9 | 0-4.0 | 15.4 | 6.7-24.2 | 15.0 | 7.9-22.0 |
|  | <$20,000 | 9.4 | 0-19.3 | 18.6 | 5.7-31.6 | 15.6 | 1.3-20.0 |

**Table S3. Weighted Prevalence and 95% Confidence Intervals (CI) of Generalized Anxiety Disorder by Demographic Features in the Metropolitan, Urban and Rural Strata.**

|  |  | **Metro** |  | **Urban** |  | **Rural** |  |
| --- | --- | --- | --- | --- | --- | --- | --- |
|  |  | ***Wtd %*** | ***95% CI*** | ***Wtd %*** | ***95% CI*** | ***Wtd %*** | ***95% CI*** |
| **Overall** |  | 4.90 | 0.7-9.1 | 6.10 | 2.9-9.3 | 10.76 | 6.1-15.5 |
| **Sex** |  |  |  |  |  |  |  |
|  | Female | **7.5** | **1.0-13.9** | **8.3** | **3.3-13.4** | **16.0** | **8.1-24.0** |
|  | Male | 1.2 | 0-3.7 | 3.2 | 0.1-6.3 | 5.0 | 1.5-8.5 |
| **Race** |  |  |  |  |  |  |  |
|  | Other | 0 | 0-0 | 1.3 | 0-3.9 | 4.6 | 0-10.5 |
|  | Black | 3.4 | 0-7.2 | 7.1 | 0-14.3 | 6.1 | 0-14.6 |
|  | White | 7.9 | 0-16.3 | 5.8 | 2.6-9.1 | 12.6 | 6.8-18.3 |
| **Ethnicity** |  |  |  |  |  |  |  |
|  | Hispanic | 4.1 | 0-12.7 |  |  | 16.1 | 0-45.7 |
|  | Non-Hispanic | 4.9 | 0.7-9.2 | 6.3 | 2.9-9.6 | 10.7 | 5.9-15.4 |
| **Age** |  |  |  |  |  |  |  |
|  | 18-29 | 0.5 | 0-1.7 | 0.4 | 0-1.1 | 14.5 | 0.5-28.5 |
|  | 30-39 | 2.2 | 0-5.5 | 10.3 | 1.8-18.9 | 23.1 | 7.1-39.0 |
|  | 40-49 | 12.5 | 0-27.2 | 6.8 | 2.0-11.6 | 4.9 | 1.6-8.2 |
|  | 50-59 | 4.5 | 0-10.1 | 6.2 | 0-14.2 | 6.3 | 1.8-10.7 |
| **Education** | |  |  |  |  |  |  |
|  | < HS | 17.4 | 0-48.5 | 10.1 | 1.8-18.5 | 7.6 | 1.7-13.4 |
|  | HS-College | 4.2 | 0-9.2 | 5.1 | 0.6-9.5 | 12.9 | 4.7-21.1 |
|  | > College | 3.3 | 0-6.7 | 3.9 | 0-7.9 | 10.0 | 3.3-16.7 |
| **Income** |  |  |  |  |  |  |  |
|  | < $20,000 | 2.5 | 0-5.5 | 12.5 | 0.5-24.4 | 10.4 | 0-22.8 |
|  | $20,-$40K | 7.5 | 0-22.9 | 5.8 | 0.5-11.1 | 18.0 | 2.6-33.5 |
|  | >$41,000 | 4.4 | 0.07-8.7 | 3.9 | 1.2-6.7 | 8.5 | 4.2-12.8 |
| **Poverty** |  |  |  |  |  |  |  |
|  | >$20,000 | 5.4 | 0-10.9 | 4.5 | 2.0-7.0 | 10.6 | 5.7-15.4 |
|  | <$20,000 | 2.5 | 0-5.5 | 12.5 | 0.5-24.4 | 10.4 | 0-22.8 |

**Table S4. Adjusted Odds Ratios for PTSD and Generalized Anxiety Disorder (GAD)**

|  | PTSD | | GAD | |
| --- | --- | --- | --- | --- |
| **Urbanicity** | Adjusted OR ^b^ | 95% CI | Adjusted OR | 95% CI |
| Rural | **2.8** | **1.2-6.9** | 0.9 | 0.3-3.2 |
| Urban | **2.9** | **1.1-7.7** | 0.6 | 0.2-2.1 |
| Metro | Referent |  | Referent |  |
| **Age** |  |  |  |  |
| 18-29 | 0.92 | 0.3-3.1 | 0.3 | 0.1-1.4 |
| 30-39 | Referent |  | Referent |  |
| 40-49 | 0.8 | 0.3-2.3 | 0.9 | 0.4-2.4 |
| 50-49 | 1.3 | 0.5-3.7 | 0.5 | 0.1-1.9 |
| **Sex** |  |  |  |  |
| Female vs. Male | **4.8** | **2.1-10.8** | **4.6** | **1.3-16.2** |
| **Race/Ethnic** |  |  |  |  |
| Other | 0.4 | 0.1-1.3 | 0.01 | 0-0.1 |
| Black | 1.3 | 0.6-2.8 | 0.2 | 0.1-0.8 |
| White | Referent |  | Referent |  |
| **Ethnic Hispanic vs. Non-Hispanic** | **8.6** | **1.8-41.8** | 3.9 | 0.7-20.4 |
| **Education** |  |  |  |  |
| < HS | **7.3** | **2.4-22.4** | 7.1 | **1.2-40.7** |
| HS-College | **3.2** | **1.1-8.8** | 2.2 | 0.8-6.3 |
| > College | Referent |  | Referent |  |
| **Poverty vs. Above Poverty** | 1.5 | 0.6-3.8 | 0.7 | 0.3-2.2 |

^a^ Adjusted odds ratios controlling for all other socio-demographic variables in the table.

^b^ Sample size of Hispanic group was small.

**Table S5. Weighted Prevalence and 95% Confidence Intervals (CI) of Moderate to Severe Depression^1^ by Demographic Features in the Metropolitan, Urban and Rural Populations.**

|  |  | **Metro** |  | **Urban** |  | **Rural** |  |
| --- | --- | --- | --- | --- | --- | --- | --- |
|  |  | ***Wtd %*** | ***95% CI*** | ***Wtd %*** | ***95% CI*** | ***Wtd %*** | ***95% CI*** |
| **Overall** |  | 7.2 | 1.9-12.5 | 10.2 | 5.2-15.3 | 6.1 | 2.9-9.2 |
| **Sex** |  |  |  |  |  |  |  |
|  | Female | 8.3 | 1.8-14.7 | 11.6 | 5.1-18.0 | 10.1 | 4.4-15.8 |
|  | Male | 5.6 | 0-14.2 | 8.5 | 0.7-16.3 | 1.7 | 0.4-2.9 |
| **Race** |  |  |  |  |  |  |  |
|  | Other | 0 | 0-0 | 0 | 0-0 | 7.8 | 0-16.9 |
|  | Black | 4.9 | 0.7-9.1 | 13.9 | 4.5-23.3 | 11.3 | 0.7-21.9 |
|  | White | 11.6 | 0.7-22.5 | 8.7 | 2.6-14.8 | 4.2 | 2.0-6.4 |
| **Ethnicity** |  |  |  |  |  |  |  |
|  | Hispanic | 4.1 | 0-12.7 | 0 | 0-0 | 2.2 | 0-6.9 |
|  | Non-Hispanic | 7.3 | 1.8-12.7 | 10.5 | 5.4-15.7 | 6.1 | 2.9-9.3 |
| **Age** |  |  |  |  |  |  |  |
|  | 18-29 | 0.7 | 0-2.1 | 6.2 | 0-16.5 | 12.9 | 0-27.5 |
|  | 30-39 | 3.3 | 0-7.3 | 15.6 | 2.8-28.4 | 8.2 | 1.6-14.6 |
|  | 40-49 | 17.9 | 0-36.1 | 12.2 | 3.8-20.5 | 3.4 | 1.1-5.7 |
|  | 50-59 | 6.8 | 0-13.7 | 6.5 | 0-14.4 | 3.3 | 1.1-5.5 |
| **Education** |  |  |  |  |  |  |  |
|  | < HS | 19.7 | 0-49.7 | 19.8 | 6.8-32.9 | 5.3 | 1.2-9.3 |
|  | HS-College | 4.1 | 0-8.5 | 7.8 | 1.9-13.7 | 7.1 | 1.7-12.4 |
|  | > College | 6.2 | 0.1-12.4 | 4.9 | 0-12.9 | 4.9 | 0-10.3 |
| **Income** |  |  |  |  |  |  |  |
|  | < $20,000 | 10.1 | 0-23.8 | 24.0 | 8.6-39.4 | 5.4 | 0.9-10.0 |
|  | $20,-$40K | 8.7 | 0-24.3 | 13.1 | 1.4-24.7 | 6.8 | 0-14.1 |
|  | >$41,000 | 5.2 | 0.6-9.8 | 4.0 | 0-8.4 | 6.2 | 1.5-10.9 |
| **Poverty** |  |  |  |  |  |  |  |
|  | >$20,000 | 6.4 | 0.6-12.1 | 6.7 | 2.1-11.4 | 6.3 | 2.3-10.3 |
|  | <$20,000 | 10.1 | 0-23.8 | 24.0 | 8.6-39.4 | 5.4 | 0.9-10.0 |

^1^Major Depressive Disorder or SDS Index Score> 60

**Table S6. Adjusted Odds Ratio for Moderate to Severe Depression** ^1^

|  | Current Moderate to Severe Depression | |
| --- | --- | --- |
| **Urbanicity** | Adjusted OR ^a^ | 95% CI |
| Rural | 0.3 | 0.1-1.2 |
| Urban | 0.7 | 0.2-2.2 |
| Metro | Referent |  |
| **Age** |  |  |
| 18-29 | 0.2 | 0.0-1.5 |
| 30-39 | Referent |  |
| 40-49 | 2.0 | 0.6-6.1 |
| 50-49 | 0.7 | 0.2-2.8 |
| **Sex** |  |  |
| Female vs. Male | 1.1 | 0.3-3.9 |
| **Race/Ethnicity** |  |  |
| Other | 0.0 | 0-0.1 |
| Black | 0.4 | 0.1-1.2 |
| White | Referent |  |
| **Ethnic Hispanic vs. Non-Hispanic** | 1.9 | 0.3-10.7 |
| **Education** |  |  |
| < HS | **5.7** | 1.2-28.0 |
| HS-College | 1.8 | 0.6-4.9 |
| > College | Referent |  |
| **Poverty vs. Above Poverty** | 2.8 | 0.6-12.7 |

^1^ Major Depressive Disorder by SCID or SDS Index Score> 60

^a^ Adjusted odds ratios controlling for all other socio-demographic variables in the table.
